# Longitudinal data on humoral response and neutralizing antibodies against SARS-CoV-2 Omicron BA.1 and subvariants BA.4/5 and BQ.1.1 after COVID-19 vaccination in cancer patients

**DOI:** 10.1007/s00432-023-04961-2

**Published:** 2023-06-10

**Authors:** Oliver Overheu, Simon Lendowski, Daniel R. Quast, Daniel Kühn, Elena Vidal Blanco, Anna-Lena Kraeft, Eike Steinmann, Eleni Kourti, Celine Lugnier, Joerg Steinmann, Anke Reinacher-Schick, Stephanie Pfaender

**Affiliations:** 1 Department of Hematology and Oncology with Palliative Care, St. Josef Hospital, Ruhr University, Bochum, Germany; 2 Department of Internal Medicine, St. Josef Hospital, Ruhr University, Bochum, Germany; 3 Department of Molecular and Medical Virology, Ruhr University, Bochum, Germany; 4 Institute of Clinical Hygiene, Medical Microbiology and Infectiology, Klinikum Nürnberg, Paracelsus Medical University, Nuremberg, Germany

**Keywords:** SARS-CoV-2, Vaccination, Omicron, BA.1, BA.4/5, BQ.1.1, Neutralizing antibodies, Cancer

## Abstract

**Purpose:**

The SARS-CoV-2 Omicron variant of concern (VOC) and subvariants like BQ.1.1 demonstrate immune evasive potential. Little is known about the efficacy of booster vaccinations regarding this VOC and subvariants in cancer patients. This study is among the first to provide data on neutralizing antibodies (nAb) against BQ.1.1.

**Methods:**

Cancer patients at our center were prospectively enrolled between 01/2021 and 02/2022. Medical data and blood samples were collected at enrollment and before and after every SARS-CoV-2 vaccination, at 3 and 6 months.

**Results:**

We analyzed 408 samples from 148 patients (41% female), mainly with solid tumors (85%) on active therapy (92%; 80% chemotherapy). SARS-CoV-2 IgG and nAb titers decreased over time, however, significantly increased following third vaccination (*p* < 0.0001). NAb (ND_50_) against Omicron BA.1 was minimal prior and increased significantly after the third vaccination (*p* < 0.0001). ND_50_ titers against BQ.1.1 after the third vaccination were significantly lower than against BA.1 and BA.4/5 (*p* < 0.0001) and undetectable in half of the patients (48%). Factors associated with impaired immune response were hematologic malignancies, B cell depleting therapy and higher age. Choice of vaccine, sex and treatment with chemo-/immunotherapy did not influence antibody response. Patients with breakthrough infections had significantly lower nAb titers after both 6 months (*p* < 0.001) and the third vaccination (*p* = 0.018).

**Conclusion:**

We present the first data on nAb against BQ.1.1 following the third vaccination in cancer patients. Our results highlight the threat that new emerging SARS-CoV-2 variants pose to cancer patients and support efforts to apply repeated vaccines. Since a considerable number of patients did not display an adequate immune response, continuing to exhibit caution remains reasonable.

## Introduction

Previous studies report an increased risk for severe coronavirus-19 disease (COVID-19) in patients with cancer and in those with weakened immune systems as compared to individuals without cancer (Lee et al. 2020; Williamson et al. 2020; Rüthrich et al. 2021). With the implementation of global vaccination strategies against severe acute respiratory syndrome coronavirus type 2 (SARS-CoV-2), vaccinating cancer patients has been shown to decrease the risk of hospitalization and mortality due to COVID-19. Moreover, early results on seroconversion following SARS-CoV-2 vaccination in cancer patients demonstrated promising results (Addeo et al. 2021; Shroff et al. 2021). However, immunogenicity was reduced compared to healthy subjects and was especially low in patients with hematological diseases or those treated with B cell depleting therapy (i.e., anti-CD20 therapy) (Giuliano et al. 2022; Nooka et al. 2022). Furthermore, titers of neutralizing antibodies (nAb) against variants of concern (VOC) were reduced compared to wild-type SARS-CoV-2 (Fendler et al. 2021; Terada et al. 2022) and levels of both IgG anti-spike (anti-S) antibodies and nAb against VOC reportedly decrease six months post vaccination (Obeid et al. 2022). Consequently, additional booster vaccinations are recommended to address this deficit (Bar-On et al. 2022; Davis-Gardner et al. 2022; Koch et al. 2022). While it has been shown that a third vaccination increases levels of IgG anti-S antibodies as well as nAb in both healthy subjects and cancer patients (Pajon et al. 2022), immune response remains low or undetectable in some patients, especially in those with B cell malignancies or anti-CD20 therapy (Lim et al. 2022). Of interest, the Omicron VOC exhibits extensive immune evasive potential (Cao et al. 2022; Cele et al. 2022; Hoffmann et al. 2022) and low or undetectable levels of nAb against this VOC were reported despite two doses of mRNA vaccines (Chang et al. 2022; Edara et al. 2022; Garcia-Beltran et al. 2022). Furthermore, Omicron subvariants like BQ.1/BQ.1.1, which has become one of the predominant VOC during winter of 2022/2023, are emerging and have demonstrated an even increased resistance to both nAb and therapeutic monoclonal antibodies (Arora et al. 2022a, b; Kurhade et al. 2022; Planas et al. 2022; Qu et al. 2022; Wang et al. 2022). However, little is known about the efficacy of a booster vaccination against the Omicron VOC in cancer patients and so far, data concerning neutralization capacity against subvariant BQ.1.1 following booster vaccination in immunocompromised patients are limited (Ehmsen et al. 2023). The present study, therefore, aims to add to the body of evidence of the impact of a third vaccination on anti-SARS-CoV-2 antibodies and their neutralizing capacity on Omicron VOC (BA.1, BA.4/5 and BQ.1.1) in a representative cohort of cancer patients.

## Methods

**Fig. 1:**
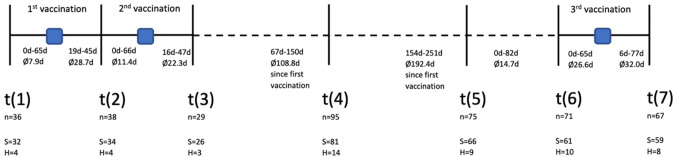
Timeline of data collection with mean time since or to vaccination and number of samples collected at each time point; t(1): prior to 1st anti-SARS-CoV-2 vaccination, t(2): after 1st vaccination, t(3): after 2nd vaccination, t(4): follow-up at 3 months, t(5): follow-up at 6 months, t(6): prior to third vaccination, t(7): after third vaccination; S: solid tumors, H: hematologic malignancies

Between 01/2021 and 02/2022, unselected cancer patients treated at our Ruhr University oncology center were prospectively included in our local COVID-19 biobanking study. All patients agreed to provide baseline medical data and multiple blood samples. Additionally, a subset of patients opted to provide further information via questionnaires (Overheu et al. 2022). As depicted in Fig. 1, sera were collected at the following time points: t(1): prior to the first vaccination, t(2): after the first vaccination, t(3): after the second vaccination, t(4): after 3 months, t(5): after 6 months, t(6): prior to third vaccination, t(7): after third vaccination. Blood serum samples were stored on-site at − 80 °C.

Laboratory analyses were performed at the Department of Molecular and Medical Virology, Ruhr University Bochum and the Institute of Clinical Hygiene, Medical Microbiology and Infectiology at Klinikum Nürnberg, Paracelsus Medical University.

The study was approved by the Ethics Committee of the Medical Faculty, Ruhr University Bochum (reference number 20-6953-bio and 21-7351) and conducted in accordance with the Declaration of Helsinki. Descriptive data are presented as *n* (%) or median (range or standard deviation). All own percentual results are rounded to the nearest full number. Data were analyzed using Fisher’s exact test, Student’s and Welch’s *t* test (depending on data variance) or *χ*^2^ test. Correlations were evaluated using Pearson’s correlation coefficient test and multivariate linear or logistic regression. Results were considered significant at *α* = 0.05. All analyses were performed using SPSS (v. 26) and GraphPad PRISM for Windows (v. 9.5.0).

### SARS-CoV-2 IgG anti-S antibodies

For the detection of SARS-CoV-2 spike-1-specific IgG concentrations, the Euroimmun enzyme-linked immunosorbent assay (ELISA) Anti-SARS-CoV-2-QuantiVac-ELISA was used according to the manufacturer’s instructions (Euroimmun AG, Lübeck, Germany). Positive and negative controls were included in each test run. Quantification of S1‐specific IgG was performed using a 6‐point calibration curve covering a range from 1 to 120 relative units (RU)/ml. By multiplication with factor 3.2, results in RU/ml were converted into standardized binding antibody units (BAU)/ml. Results < 25.6 were considered negative, ≥ 25.6–< 35.2 borderline, and ≥ 35.2 positive.

### SARS-CoV-2 neutralizing antibody ELISA

To determine nAb against parental (wildtype) SARS-CoV-2-Spike in patient sera, a competitive ELISA Kit from Invitrogen (#BMS2326) was used. The ELISA was performed and evaluated according to manufacturer instructions. Briefly, receptor binding domain (RBD) pre-coated ELISA plates were incubated with patient sera to specifically bind nAb. Afterwards, the plates were incubated with biotinylated ACE2. Streptavidin-HRP conjugate was used with a chromogene substrate to detect ACE2-RBD binding with a plate reader (Berthold). ELISA results were used to select the patient cohort for the following determination of neutralizing antibody titers (PVND_50_) for Anti-SARS-CoV2-S antibodies against Omicron subvariants BA.1 (B 1.1.529), BA.4/5 and BQ1.1 (sera included with neutralizing capacities > 20% in the ELISA).

### SARS-CoV-2 neutralization pseudovirus assay

Expression plasmids harboring the pCG1-SARS-CoV- 2 BA.1 (B 1.1.529) SΔ18 (codon-optimized, C-terminal truncation of 18 amino acid residues, GISAID Accession ID: EPI_ISL_6640919), pCG1-SARS-CoV-2 BA.4/5 SΔ18 (codon-optimized, C-terminal truncation of 18 amino acid residues, GISAID Accession ID: EPI_ISL_11550739 and EPI_ISL_12029894) and SARS-CoV-2 BQ.1.1 SΔ18 (GISAID Accession ID: EPI_ISL_14752457) were kindly provided by S. Pöhlmann and M. Hoffmann (German Primate Center-Leibniz Institute for Primate Research, Göttingen, Germany) and have been described before (Arora et al. 2022a, b; Hoffmann et al. 2022). VSV*∆G(FLuc)-pseudoparticles, harboring the BA.1, BA.4/5 or BQ.1.1 spike were produced as previously described (Zettl et al. 2020). Briefly, the rhabdoviral pseudotyped particles were produced in 293 T cells transfected to express the Omicron SARS-CoV-2-S subvariants and inoculated with VSV*∆G-FLuc, a replication-deficient vesicular stomatitis virus (VSV) vector that encodes for enhanced green fluorescent protein and firefly luciferase (FLuc) instead of VSV-G protein (kindly provided by Gert Zimmer, Institute of Virology and Immunology, Mittelhaeusern, Switzerland). Pseudoparticles were collected, cleared from cellular debris by centrifugation and stored at − 80 °C until further use. For the virus neutralization assay, sera were incubated for 30 min at 56 °C to inactivate complement factors. SARS-CoV-2 pseudoparticles were incubated with triplicates of twofold serial dilutions of immune sera in 96-well plate prior to infections of Vero E6 cells (1 × 10^4^ cells/well) in DMEM + 10% FBS (Life Technologies). At 18 h post infection, firefly luciferase (FLuc) reporter activity was determined and the reciprocal antibody dilution causing 50% inhibition of the luciferase reporter calculated (PVND_50_; lower limit of detection: 20 PVND_50_; upper limit of detection: 2560 PVND_50_). Analysis for nAb against subvariants BA.4/5 and BQ.1.1 was only performed for sera collected after the third vaccination.

## Results

### Patient characteristics

A total of 148 patients (41% female) were included. Baseline characteristics are presented in Table 1. Mean age was 63.9 (24–87) years. Most patients suffered from solid tumors (*n* = 126, 85%), mainly gastrointestinal (*n* = 87, 59%). The most frequent diagnosis was pancreatic cancer (*n* = 59, 40%). Most patients were on active cancer therapy (*n* = 136, 92%), mainly chemotherapy (*n* = 118, 80%), only four (3%) patients received B cell-depleting therapy. History of COVID-19 was present in seven (5%) patients and five (3%) patients were diagnosed with COVID-19 during the study (one patient after basic immunization and four patients after third vaccination).

**Table 1:** Baseline characteristics of the participants

Parameter	All participants	Non-hematologic malignancies	Hematologic malignancies	Significance of differences
(*p* value)				
N (% of total)	148 (100%)	126 (85%)	22 (15%)	
Female (% female)	61 (41%)	52 (41%)	9 (41%)	0.58
Age (years)	64 (24–87)	64 (± 11.5)	62 (± 16.5)	0.46
BMI	25.0 (17.2–41.3)	24.8 (± 4.2)	26.4 (± 4.9)	0.11
Active therapy	136 (92%)	118 (94%)	18 (82%)	0.08
Chemotherapy	118 (80%)	104 (83%)	14 (64%)	**0.046**
Immunotherapy	23 (16%)	20 (16%)	3 (14%)	0.54
Targeted therapy	20 (14%)	16 (13%)	4 (18%)	0.34
Radiation	20 (14%)	18 (14%)	2 (9%)	0.4
B Cell depleting therapy	4 (3%)	0 (0%)	4 (18%)	** < 0.001**
Mean duration of disease at first vaccination (months)	27 (0–243)	29 (± 44.4)	21 (± 24.9)	0.56
Basic anti-SARS-CoV-2 immunization		117/136 (86%)	19/136 (14%)	
BNT162b2	115/136 (85%)	98/117 (84%)	17/19 (89%)	–
mRNA-1273	1/136 (1%)	1/117 (1%)	0 (0%)	–
ChAdOx1-S	18/136 (13%)	16/117 (14%)	2/19 (11%)	–
Ad26.COV2.S	2/136 (2%)	2/117 (2%)	0	–
Unknown	12 (8%)	9	3	–
Third anti-SARS-CoV-2 vaccination	95 (64%)	80 (63%)	15 (68%)	0.81
BNT162b2	76 (80%)	65/80 (81%)	11/15 (73%)	–
mRNA-1273	19 (20%)	15/80 (19%)	4/15 (27%)	–
History of COVID-19	7 (5%)	7 (6%)	0 (0%)	0.29
History of influenza vaccination within five years	73 (49%)	63 (50%)	10 (45%)	0.62
Use of NSAID, Paracetamol or dexamethasone following initial vaccination (up to 3 days)	13/88 (15%)	10/76 (13%)	3/12 (25%)	0.38

Data are presented as *n* (%) or mean (range). Differences in number are due to not all patients answering the related questionnaire. Significance was determined using Student’s *t* test or *χ*^2^ test

Most patients had BNT162b2 (85%) for their basic immunization. In total, 95 (64%) patients received a third SARS-CoV-2 vaccination (*n* = 76 (80%) BNT162b2, *n* = 19 (20%) mRNA-1273).

### Humoral immune responses following vaccination

**Fig. 2:**
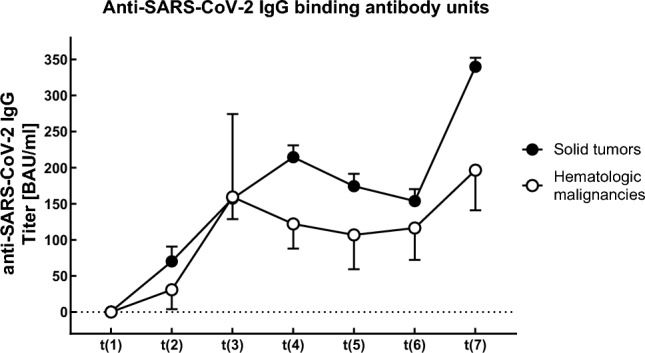
Course of mean anti-SARS-CoV-2 IgG in cancer patients with solid tumors or hematologic malignancies over the course of the study

We analyzed a total of 408 serum samples (Fig. 1). Analysis of anti-SARS-CoV-2 IgG binding antibody units (BAU) demonstrated significantly increased antibody titers following the third vaccination compared with all other time points (*p* < 0.0001). Antibody levels were significantly lower following the first SARS-CoV-2 vaccination (*p* ≤ 0.009), except compared with the respective levels prior to the third vaccination (*p* = 0.076), indicating a decline in antibody titers over time (Fig. 2).

**Fig. 3:**
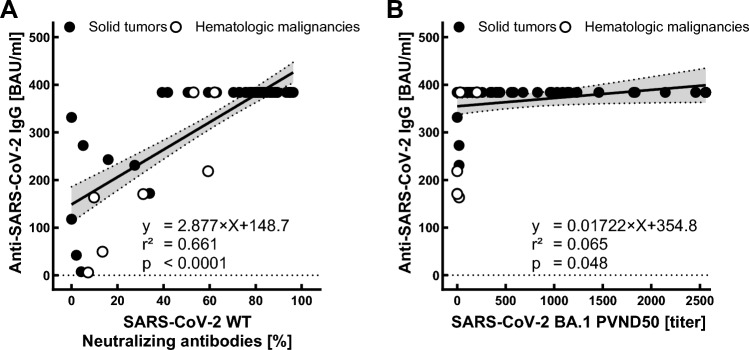
Correlations of anti-SARS-CoV-2 IgG with parental SARS-CoV-2 neutralizing antibody (nAb) titers and SARS-CoV-2 Omicron BA.1 neutralization titers (ND_50_) following third vaccination

Following the third anti-SARS-CoV-2 vaccination, the proportion of neutralizing antibodies (nAb) against parental SARS-CoV-2 (wild-type) increased significantly (153.8 vs. 339.7 BAU/ml, *p* < 0.0001). Overall, 85% of patients elicited nAb with a neutralizing capacity > 20% after booster vaccination, compared to only 52% after 3 months (*p* < 0.001). The titers of nAb against wild-type SARS-CoV-2 after the third vaccination were significantly correlated with anti-SARS-CoV-2 IgG BAU titers (*r* = 0.813, *p* < 0.0001; Fig. 3A). ND_50_ titers against the Omicron subvariant BA.1 were significantly higher following third vaccination (*p* < 0.0001) and overall significantly correlated with anti-SARS-CoV-2 IgG antibody levels (*r* = 0.239, *p* < 0.0001; Fig. 3B) and proportion of nAb against parental (wild-type) SARS-CoV-2 (*r* = 0.3; *p* < 0.0001).

**Fig. 4:**
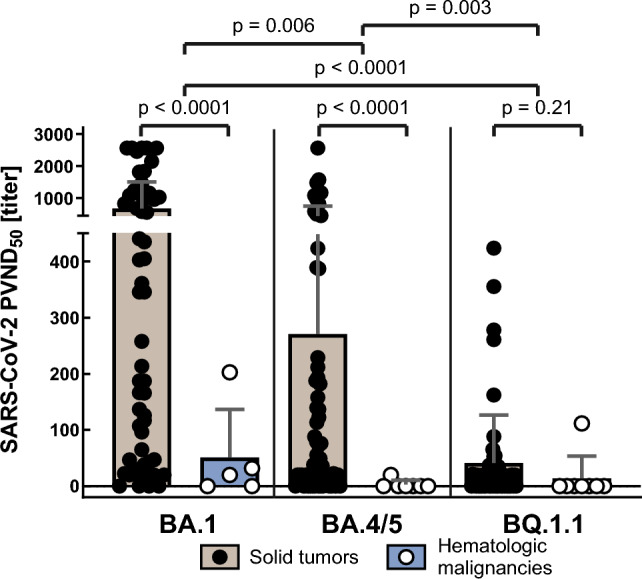
Neutralizing antibody titers (PVND_50_) against SARS-CoV2 Omicron subvariants following third vaccination (t(7)) in cancer patients

However, while ND_50_ titers after the third vaccination correlated significantly with their corresponding BAU titer levels after the third vaccination (*r* = 0.254, *p* = 0.048), they did not correlate with BAU levels at any other individual time point. ND_50_ titers against Omicron subvariants BA.4/5 and BQ.1.1 after third vaccination (*n* = 65) tended to correlate with corresponding BAU titers (BA.4/5: *r* = 0.22, *p* = 0.076; BQ.1.1: *r* = 0.187, *p* = 0.136) and significantly correlated with corresponding nAb titers against parental SARS-CoV-2 (BA.4/5: *r* = 0.29, *p* = 0.019; BQ.1.1: *r* = 0.268, *p* = 0.031). There were significant differences between mean ND_50_ titers against BA.1, BA.4/5 and BQ.1.1, with the latter being significantly lower compared to the former (36.78 vs. 241.3 vs. 621.3, *p* < 0.0001; Fig. 4). Nearly half of the patients evaluated for nAb against BQ.1.1 (31/65, 48%) and all evaluated patients with a hematologic malignancy did not demonstrate any detectable titer level.

Choice of booster SARS-CoV-2 vaccine did neither significantly influence nAb (BNT162b2 vs. mRNA-1273: 64% vs. 71%; *p* = 0.45) nor ND_50_ titers post-third vaccination (*p* = 0.43).

BAU titers (*p* = 0.008) and expression of nAb (32% vs. 58%; *p* = 0.002) were significantly higher among those with a history of COVID-19, while ND_50_ titers did not differ (*p* = 0.28). Patients with SARS-CoV-2 breakthrough infections had significantly lower BAU and nAb titers against parental SARS-COV-2, both at 6 months follow-up (12.1 vs. 179.6 BAU/ml, *p* < 0.001; 0% vs. 26%, *p* < 0.001) and after third vaccination (196.5 vs. 347.7 BAU/ml, *p* = 0.011; 29% vs. 69%, *p* = 0.018). The characteristics of the patients with breakthrough infections are displayed in Table 2.

**Table 2:** Characteristics of the patients with SARS-CoV-2 breakthrough infections

No	1	2	3	4	5
Time of infection	After basic immunization	After 3rd vaccination	After 3rd vaccination	After 3rd vaccination	After 3rd vaccination
Malignancy	Pancreatic cancer	Myeloma	Pancreatic cancer	Pancreatic cancer	Lung cancer
Age	71	74	83	39	73
Sex	Male	Male	Female	Male	female
Therapy	CTx	Targeted therapy	None	CTx	CTx
Disease activity	Uncontrolled	Controlled	Remission	Controlled	Uncontrolled
COVID-19 severity	Mild to moderate	Mild to moderate	Mild	Mild	Mild to moderate
Third vaccine	–	BNT162b2	BNT162b2	BNT162b2	BNT162b2
BAU/ml at 6 months follow-up	–	13.4	10.8	–	–
nAb % at 6 months follow-up	–	0	0	–	–
BAU/ml post-third vaccination	–	163.5	42.2	–	384
nAb % post-third vaccination	–	10	2	–	76

BAU, binding antibody units; nAb, neutralizing antibodies against parental SARS-CoV-2; CTx, chemotherapy; BNT, BNT162b2

### Influence of cancer entity and therapy

**Fig. 5:**
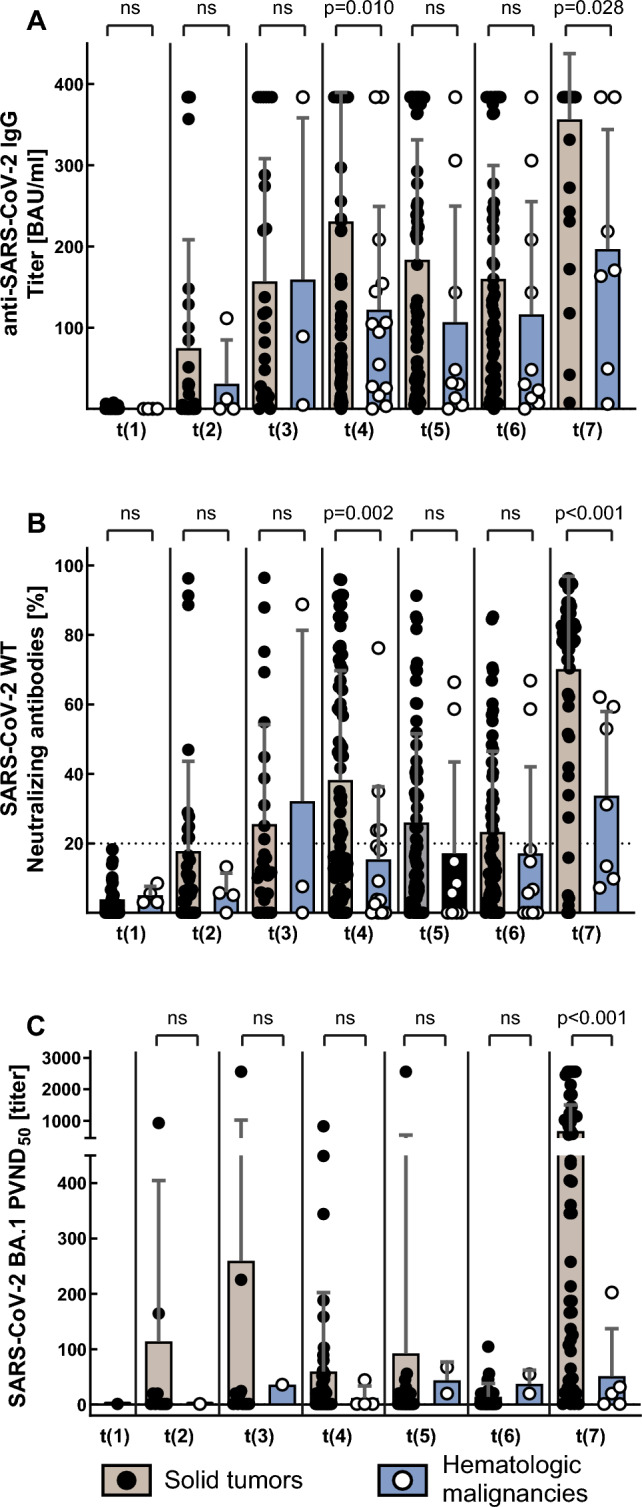
Titers for anti-SARS-CoV-2 IgG, SARS-CoV-2 parental neutralizing antibodies and SARS-CoV-2 Omicron BA.1 PVND_50_ in cancer patients with solid tumors or hematologic malignancies

Mean titers of BAU, nAb and ND_50_ were significantly lower in patients with hematologic diseases than in those with solid malignancies, both over all time points (197.9 vs. 103.3 BAU/ml; *p* < 0.0001; nAb: 17% vs. 35%, *p* < 0.0001; ND_50_: 38.4 vs. 275.1, *p* < 0.0001) and following third vaccination (196.6 vs. 356.4 BAU/ml, *p* = 0.028; nAb: 34% vs. 70%, *p* < 0.001; ND_50_: 50.9 vs. 672.2, *p* < 0.0001; Fig. 5). This also applied to ND_50_ titers against Omicron subvariant BA.4/5, but not BQ.1.1 after third vaccination (BA.4/5: 2.9 vs. 270.5, *p* < 0.001; BQ.1.1: 0 vs. 41.2, *p* < 0.001; Fig. 4).

**Fig. 6:**
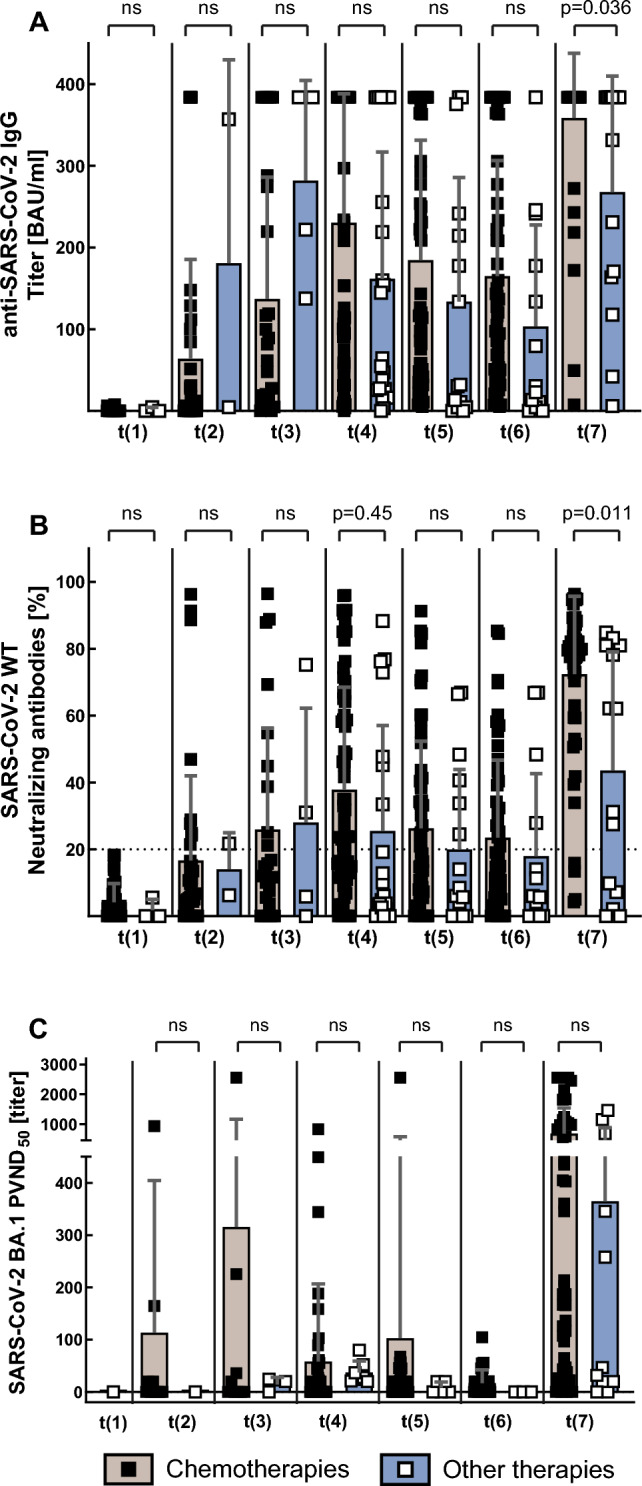
Titers for anti-SARS-CoV-2 IgG, parental SARS-CoV-2 neutralizing antibodies and SARS-CoV-2 Omicron BA.1 PVND_50_ in cancer patients treated with chemotherapy or other therapies

Patients receiving B cell-depleting therapy had significantly lower BAU levels (63.4 vs. 190.6 BAU/ml; *p* < 0.0001). Accordingly, nAb titers were also significantly lower among patients receiving B cell-depleting therapy (9% vs. 33%; *p* < 0.0001). Treatment with chemotherapy (192.1 vs. 168.0 BAU/ml, *p* = 0.22) or immunotherapy (193.3 vs. 186.8 BAU/ml; *p* = 0.75) did not significantly influence antibody response over all time points, except for BAU and nAb against parental SARS-CoV-2 levels after the third vaccination, where titers were significantly higher among patients receiving chemotherapy (Fig. 6A/B).

### Influence of various clinical factors

**Fig. 7:**
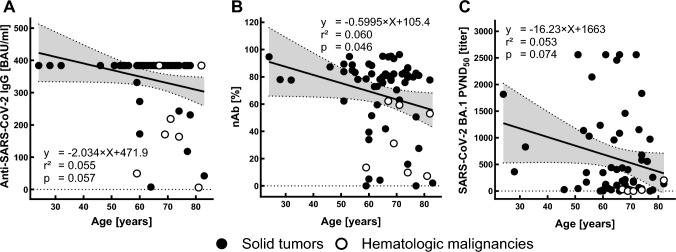
Correlations of anti-SARS-CoV-2 IgG, parental SARS-CoV-2 neutralizing antibody titers (nAb) and SARS-CoV-2 Omicron neutralization titers (ND_50_) with age after third vaccination

BAU and nAb titers were overall significantly negatively correlated with patients age (*r* =  − 0.122, *p* = 0.01; *r* =  − 0.128; *p* = 0.007). After the third vaccination titers of nAb against wild-type SARS-CoV-2 were significantly correlated with age (*r* =  − 0.245, *p* = 0.046; Fig. 7B), while BAU levels and titers against Omicron subvariants showed a non-significant trend (BAU: *r* =  − 0.234, *p* = 0.057; BA.1: *r* =  − 0.23, *p* = 0.074; BA.4/5: *r* =  − 0.21, *p* = 0.096; BQ.1.1: *r* =  − 0.11, *p* = 0.4; Fig. 7A/C). Antibody titers also showed a partially significant correlation with patients BMI (body mass index; BAU: *r* = 0.09, *p* = 0.064; nAb: *r* = 0.123; *p* = 0.011; ND_50_: *r* = 0.143, *p* = 0.038; ND_50_ BA.4/5: *r* = 0.322, *p* = 0.011). No sex-specific differences were detected (BAU: *p* = 0.25; nAb: 0.993; ND_50_: *p* = 0.83).

Use of non-steroidal anti-inflammatory drugs (NSAID), paracetamol or dexamethasone on the day of or following the initial or second vaccination neither influenced anti-SARS-CoV-2 IgG nor nAb levels.

Interestingly, patients who had an influenza vaccination within five years prior displayed a significantly higher SARS-CoV-2 IgG anti-S antibody and nAb response following their initial vaccination (114.3 vs. 25.1 BAU/ml, *p* = 0.05; 28% vs. 8%, *p* = 0.035), but not after third vaccination or any other timepoint of the study. Prior pneumococcal vaccination did not influence antibody response.

Out of those patients who were evaluated post-booster vaccination, six (6/61, 10%) did not exhibit detectable ND_50_ titers. These patients had significantly lower neutralization capacities against parental SARS-CoV-2 (39% vs. 73%; *p* = 0.001) and lower BAU titers, although slightly not significant (*p* = 0.064). Their characteristics are displayed in Table 3.

**Table 3:** Characteristics of the patients with ND_50_ titers against SARS-CoV-2 Omicron BA.1 below lower detection limit

No	1	2	3	4	5	6
Malignancy	Leukemia	Leukemia	Pancreatic cancer	Pancreatic cancer	Pancreatic cancer	Head and neck cancer
Age	69	71	77	60	66	59
Sex	Male	Male	Male	Female	Male	Female
Therapy	none	CTx	CTx	CTx	CTx	IO
Disease activitiy	Uncontrolled	Controlled	Remission	Controlled	Controlled	Controlled
Third vaccine	UNK	mRNA-1273	BNT	BNT	BNT	BNT
Days since third vaccination	27	12	52	34	19	29
BAU/ml post-third vaccination	170.6	218.6	384	172.1	384	331.5
nAb % post third vaccination	31	59	51	34	42	19

BAU, binding antibody units; nAb, neutralizing antibodies against parental SARS-CoV-2; CTx, chemotherapy; IO, immunotherapy; UNK, unknown; BNT, BNT162b2

## Discussion

This study presents data on humoral immune response in a representative cohort of cancer patients vulnerable to SARS-CoV-2 infections and severe COVID-19, including antibody analysis and neutralization titers of the currently most important and emerging variants of concern (i.e., Omicron BA.1, BA.4/5 and BQ.1.1). Most of the current studies on antibody response against the Omicron VOC focus on specific subgroups (e.g., lung cancer patients Mack et al. 2022; Valanparambil et al. 2022), patients with B cell malignancies (Greenberger et al. 2021) or patients after allogeneic hematopoietic stem cell transplant (Canti et al. 2022; Watanabe et al. 2022). So far, there are only a few reports on the efficacy of a third vaccination comparing different malignancies and treatments (Lasagna et al. 2022; Shapiro et al. 2022a, b; Zeng et al. 2022), however, those demonstrate an increase in antibody titers and nAb against Omicron after booster vaccination (Fendler et al. 2022). Nonetheless, questions remain as some patients still exhibit low immune responses. Furthermore, the durability of immunization, especially against VOC, is still not clear, necessitating additional studies with regard to subvariants and the influence of various factors like applied vaccine, age, cancer type, treatments or sex.

Based on the results of the IgG antibody titers, our study provides longitudinal data demonstrating an adequate immune response in most patients with cancer in regards of antibody titers. However, while antibody levels correlated with the relevant outcome of neutralizing antibodies against parental SARS-CoV-2, a considerable number of patients exhibited low levels of nAb three months following initial vaccination. Those increased significantly following the third vaccination. This is in line with previous data on neutralizing antibodies against SARS-CoV-2 VOCs and the efficacy of the administration of a booster vaccine (Khan et al. 2022; Terada et al. 2022; Wagner et al. 2022). Rate of SARS-CoV-2 breakthrough infections in our cohort was low. However, those patients who contracted COVID-19 post-vaccination had significantly lower antibody and nAb titers prior to their infection, indicating both vaccine effectiveness in the remaining cohort as well as an association of reduced antibody responses with a higher risk of SARS-CoV-2 breakthrough infections. This is supported by recent data from Lee et al. and highlights the importance of additional vaccinations for groups at risk (Lee et al. 2022; Macrae et al. 2022). None of our patients with breakthrough infection experienced a severe course of COVID-19.

In addition, ND_50_ titers against the Omicron VOC BA.1 were minimal prior and increased significantly after the third vaccination. We demonstrated ND_50_ levels to be independent of SARS-CoV-2 IgG titers and nAb against the parental SARS-CoV-2 strain, except for post-third vaccination levels. However, a concerning number of patients in our cohort (10%) did not elicit detectable ND_50_ titers against BA.1 after booster vaccination, although BAU and nAb titers were high. Only a third of these patients had a hematological malignancy and were therefore considered severely immune impaired, the remaining patients had solid cancers and received conventional cytostatic chemotherapy or immunotherapy. Low or undetectable neutralization of Omicron BA.1 after anti-SARS-CoV-2 vaccination in cancer patients has previously been described (Garcia-Beltran et al. 2022; Terada et al. 2022). This highlights the Omicron VOC’s capability of immune evasion as well as the need for the administration of a booster vaccine in cancer patients to cope with this. Further research is required to explain impaired immune responses in oncological patients, especially with respect to evasive VOCs like Omicron and its subvariants (Valanparambil et al. 2022; Zeng et al. 2022).

To the authors’ knowledge, this is one of the first studies providing data on neutralization concerning the Omicron BQ.1.1 subvariant following the third vaccination in a cohort of cancer patients. ND_50_ titers against BQ.1.1 were significantly lower than those against BA.1 or BA.4/5, which is coherent with recent results by Ehmsen et al. (2023). Almost half of our evaluated patients did not elicit detectable titers. Together with this subvariant’s previously reported resilience against monoclonal antibodies, this finding highlights the considerable risk of this and potentially other emerging SARS-CoV-2 to cancer patients.

In accordance with previous findings, SARS-CoV-2 IgG and nAb levels decreased over time and were negatively correlated or associated with patients’ age, a hematological malignancy or treatment with B cell depleting therapy, respectively (Lim et al. 2022; Obeid et al. 2022; Shapiro et al. 2022a, b). Choice of mRNA booster vaccination or sex did not influence the observed immune response.

Interestingly, we found a prior influenza vaccination to be significantly correlated to increased levels of nAb following the initial SARS-CoV-2 vaccination in cancer patients. Potentially, this could be explained by cross-immunity or trained immunity, as previously reported for SARS-CoV-2 infected patients with prior influenza vaccination (Debisarun et al. 2021; Poniedzialek et al. 2022). More research is necessary to fully elucidate this aspect, as this might indicate an interesting mechanism to increase immune response in cancer patients or potentially other patients with an impaired immune system.

Some limitations need to be considered: T-cell responses were not analyzed and, albeit data on healthy groups and reduced immunogenicity or efficacy of anti-SARS-CoV-2 vaccination in cancer patients has been previously reported (Garcia-Beltran et al. 2022; Gong et al. 2022; Ozbay Kurt et al. 2022; Pajon et al. 2022; Valanparambil et al. 2022), the study lacks a healthy control group.

In conclusion, our research provides extensive longitudinal data on cancer patient’s immune response and associated clinical factors following SARS-CoV-2 vaccination with a focus on the third (“booster”) vaccination. These are also among the first data on protection against the emerging Omicron subvariant BQ.1.1 in a representative cohort of cancer patients. Since a considerable number of patients did not display an adequate immune response, our results support continued efforts to apply booster and possibly repeated vaccines as well as reasonable caution. Further research is warranted to identify underlying mechanisms to overcome impaired immune response.

## Data Availability

The data used and analyzed during this study are available from the corresponding author upon reasonable request.
